# The impacts of artificial light at night on the ecology of temperate and tropical reefs

**DOI:** 10.1098/rstb.2022.0362

**Published:** 2023-12-18

**Authors:** Emily K. Fobert, Colleen R. Miller, Stephen E. Swearer, Mariana Mayer-Pinto

**Affiliations:** ^1^ School of BioSciences, University of Melbourne, Parkville, Victoria 3010, Australia; ^2^ Department of Ecology and Evolutionary Biology, Cornell University, Ithaca, NY 14853, USA; ^3^ National Centre for Coasts and Climate (NCCC), School of BioSciences, University of Melbourne, Parkville, Victoria 3010, Australia; ^4^ Centre for Marine Science and Innovation, Evolution and Ecology Research Centre, School of Biological, Earth and Environmental Science, University of New South Wales, Sydney, New South Wales 2052, Australia

**Keywords:** light pollution, artificial light at night, temperate reefs, coral reefs, trophic interactions, anthropogenic stressor

## Abstract

Despite 22% of the world's coastal regions experiencing some degree of light pollution, and biologically important artificial light at night (ALAN) reaching large portions of the seafloor (greater than 75%) near coastal developments, the impacts of ALAN on temperate and tropical reefs are still relatively unknown. Because many reef species have evolved in response to low-light nocturnal environments, consistent daily, lunar, and seasonal light cycles, and distinct light spectra, these impacts are likely to be profound. Recent studies have found ALAN can decrease reproductive success of fishes, alter predation rates of invertebrates and fishes, and impact the physiology and biochemistry of reef-building corals. In this paper, we integrate knowledge of the role of natural light in temperate and tropical reefs with a synthesis of the current literature on the impacts of ALAN on reef organisms to explore potential changes at the system level in reef communities exposed to ALAN. Specifically, we identify the direct impacts of ALAN on individual organisms and flow on effects for reef communities, and present potential scenarios where ALAN could significantly alter system-level dynamics, possibly even creating novel ecosystems. Lastly, we highlight large knowledge gaps in our understanding of the overall impact of ALAN on reef systems.

This article is part of the theme issue ‘Light pollution in complex ecological systems’.

## 1. Introduction

Light pollution, or artificial light at night (ALAN), is increasingly considered one of the most pervasive forms of global environmental change [1]. A large proportion of the planet is now exposed to artificially lit skies at night, and the global extent and pervasiveness of ALAN is increasing at a rate of more than 9% per year [2]. This is concerning for nearly all life on the Earth, as most organisms have evolved in reliable light-dark environments, with daily, lunar and seasonal light cycles that regulate critical biological processes [3].

Marine environments are not exempt from this largely land-based pollutant. Both direct sources of light (e.g. from marine infrastructure, coastal lighting) and skyglow (lighting emitted or reflected upwards, scattered in the atmosphere and reflected back to the Earth's surface; [4]) from urban centres contribute to the artificial light that can disrupt marine life in coastal habitats. In fact, ALAN from coastal developments reaches more than 22% of the world's near-shore environments [5]. An estimated 1.6 million km^2^ of the world's coastal seas are exposed to ALAN at a depth of 10 m [6], and biologically important levels of light are likely to reach depths of up to 70 m (from artificial sky glow) to 100 m (from e.g. waterside street lighting; [7]). Rapid urbanization of global coastlines [8] means that ALAN in shallow marine environments, where most of the world's marine biodiversity is found [9], is expected to continue to increase in extent and intensity [10].

This widespread exposure of marine systems to ALAN is of significant ecological concern for two reasons. First, the predictability and magnitude of changes in nocturnal illumination throughout the lunar cycle has led to many organisms on coral and temperate reefs evolving biological rhythms that change with variation in moonlight [11,12]. Consequently, moonlight, and/or the lack of (i.e. darkness), is a critical time cue for the proper functioning of reef ecosystems, and is intrinsically tied to reproduction, settlement and recruitment, activity and trophic interactions on shallow reefs [11]. Even small changes in the light environment are detectable by many marine organisms and have the potential to significantly impact biological or ecosystem function. Second, the impacts of ALAN exposure are likely to be amplified by the relatively recent move to energy efficient, broadband light-emitting diodes (LEDs; [7]), which are rich in short wavelengths. Short wavelengths penetrate deeper through the water column, and many marine organisms are naturally sensitive to these short wavelengths owing to the environment they have evolved in [7,13]. Therefore, growing global use of LEDs is likely to increase the exposure and impacts of ALAN on marine systems, including shallow coral and temperate reefs.

Despite the extensive reach of ALAN in the marine environment and the likelihood of significant disruption to shallow reef systems, research on the impacts of ALAN on tropical and temperate reefs is still minimal and focuses almost exclusively on impacts at the organism level. How changes to individual species fitness, behaviour and circadian rhythms caused by exposure to ALAN scale up to shape population, community and system-level dynamics on reefs is still largely unknown. In this paper, we integrate knowledge of the role of natural light in temperate and tropical reefs with a synthesis of the current literature on the impacts of ALAN on reef organisms to explore potential changes at the system level in reef communities exposed to light pollution. In the tropics, we focus on coral reefs, while in temperate regions, ‘reefs' include rocky reefs, as well as those formed by habitat-forming organisms such as seaweeds (e.g. kelp forests), bivalves (e.g. oyster reefs) and annelids (e.g. *Sabellaria* spp. reefs), where relevant information is available. Specifically, we discuss the potential mechanisms of how ALAN can drive reef ecosystem change through direct impacts on individual organisms and flow on effects—the secondary or indirect consequences to interconnected species or processes—for reef communities (§2). We then present potential scenarios where ALAN could significantly alter system-level dynamics, possibly even creating novel ecosystems (§3). Lastly, we discuss key knowledge gaps and directions for future research on the consequences of ALAN for temperate and tropical reefs (§4).

## 2. Direct and indirect mechanisms of change under artificial light at night

**Figure 1.**
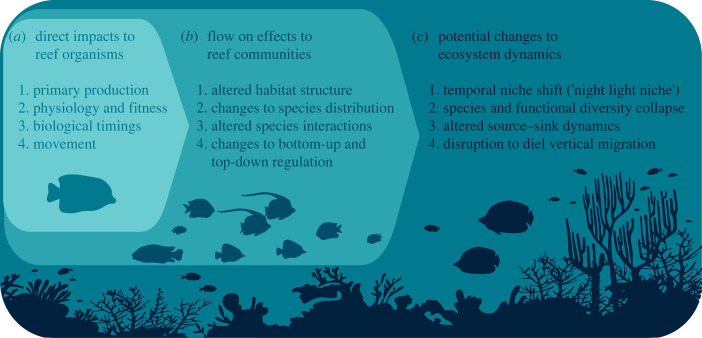
As a stressor, ALAN can simultaneously directly impact species across all trophic levels, and multiple levels of biological organization (individual, community, ecosystem). In this paper, we discuss how known impacts of ALAN to (*a*) primary production, physiology and fitness, biological timings, and movement behaviour can scale up to re-shape reef communities by (*b*) altering the physical structure of the reef, redistributing species among reef habitat, disrupting or shifting the outcomes of species interactions, and shifting the balance of bottom-up and top-down regulation on the reef. These changes to reef communities have the potential to significantly alter ecosystem dynamics, possibly even creating novel ecosystems. Potential scenarios where ALAN could create novel ecosystems include (*c*) creation of the ‘night light niche’, causing diversity collapse of key reef organisms, interfering with population connectivity and source–sink dynamics, and disrupting the complex trophic interactions during the diel vertical migration. (Online version in colour.)

ALAN is an anthropogenic disturbance that can simultaneously directly affect all trophic levels, with indirect effects to higher and lower trophic levels through bottom-up and top-down processes. As a result of these direct and cascading impacts, ALAN has the potential to significantly alter community structure and whole system function. Here, we have identified four key mechanisms or processes driven by natural light conditions, by which ALAN can alter reef ecosystems through direct and indirect impacts on individual organisms: (i) by increasing primary production; (ii) through impacts on the physiology, reproduction, and survival of reef community members; (iii) by interfering with biological timings of key events; and (iv) by influencing the movement of organisms through phototactic behaviour (attraction/repulsion) (figure 1*a*). These four mechanisms are not mutually exclusive, and in fact, probably co-occur on reefs creating a feedback loop in which ALAN can simultaneously directly and indirectly influence each mechanism. Changes resulting from any one of these mechanisms will probably have cascading effects through the food-web, re-shaping reef communities (figure 1*b*), and potentially creating novel ecosystem dynamics (figure 1*c*). In this section, we explore these mechanisms further by first discussing the importance of nocturnal illumination for each underlying process, and second, by providing the evidence, or the potential, for direct and indirect effects of ALAN on individual organisms and outcomes for reef communities.

### (a) Primary production

Primary production is a core process underpinning the functioning of coral [14] and temperate reefs [15]. Impacts to primary production can result in the gain or loss of primary habitat, altered production and assimilation of biomass, and transfer of nutrients to higher trophic levels within the reef system [14]. The magnitude of primary production is largely driven by the light intensity in the environment [16], as increases in light irradiance on a reef can enhance the photosynthetic activity and growth of photoautotrophs (e.g. biofilms, micro and macroalgae, and corals and their endosymbiotic algae; [17–19]).

While little research to date has specifically assessed the impacts of ALAN on primary productivity on subtidal reefs, the addition of light to the reef environment will probably lead to increased primary productivity of the system. Studies on terrestrial plants confirm that relatively low levels of short durations of ALAN can effectively influence the growth and reproduction of terrestrial autotrophs [20]. Similarly, ALAN from white LED lighting has been shown to significantly increase maximum photosynthetic efficiency and photosynthetic biomass of epilithic microphytobenthos on rocky intertidal shorelines [21], and increase biomass and alter assemblage diversity of phytoplankton communities [22]. Therefore, by increasing the availability of light for photosynthesis, ALAN probably enhances primary productivity on coral and temperate reefs. Increased growth and productivity have bottom-up effects on the system, as more nutrients become available for higher trophic levels. However, the net impact of ALAN on primary production is not necessarily positive. ALAN can also reduce primary production on a reef indirectly through influencing top-down effects, i.e. increasing grazing pressure on primary producers. For example, in a temperate rocky intertidal food-web, Maggi & Benedetti-Cecchi [21] found increased consumption of epilithic microphytobenthos by herbivorous gastropods compensated for the positive effect of ALAN on primary productivity. Other studies, however, found no effects of ALAN on consumption rates of grazers, such as sea-urchins [23], highlighting the fact that responses to ALAN are species and context specific, and emphasizing the need to move beyond single organism studies in assessing the impacts of ALAN on trophic interactions, and community and ecosystem level dynamics.

### (b) Physiology, fitness and survival

Light is a critical environmental cue for the regulation of key hormone pathways responsible for the physiological functioning of an individual. In both vertebrates [24] and invertebrates [25], light is the primary environmental cue regulating the synthesis of melatonin, a key hormone involved in the regulation of circadian rhythms [24]. In a large range of species, the synthesis and release of melatonin occurs in darkness and is suppressed during daylight hours. Suppression or alteration of melatonin synthesis owing to changes in the night light environment can directly impact sleep/rest periods, and can have cascading effects on other hormonal pathways, including those affecting metabolism and energy expenditure, reproductive timing and fitness, stress, oxidative damage and survival in vertebrates [24], as well as moulting and metamorphosis in invertebrates [25].

On coral reefs, Scleractinian or hard, corals are critical to the structure and resilience of the reef ecosystem, and corals and their symbionts are highly photosensitive [26]. Corals are adapted to the natural light present in their environment, and coral growth, photosynthetic activity and health is dependent on both irradiance and wavelength of light (e.g. deeper corals exhibit higher photosynthetic rates in blue, short-wavelength light, compared to shallow-living corals which have higher photosynthetic rates in broad-spectrum lighting; [27]). The light and dark diel cycle is also critical for stress recovery and repair of coral photosynthetic symbionts [28,29], and therefore the presence of ALAN can erode these relationships. Indeed, ALAN has been found to elicit oxidative stress and reduce photosynthetic performance in Red Sea corals [28,30], with impacts found to be more extreme under shorter wavelength light (blue and white spectrum LED lights compared to yellow spectrum LED lights; [30]). ALAN can also disrupt the coral-dinoflagellate symbiosis [28,31], and alter pathways of gene expression, with approximately 25 times more differentially expressed genes that regulate cell cycle, cell proliferation, cell growth and protein synthesis under ALAN [26]. Furthermore, ALAN can affect gametogenesis and the timing of gamete release, impacting coral reproduction ([32]; see §2c Biological timings). Though research on the physiological impacts of ALAN on corals is only in its infancy, these studies demonstrate the potential for ALAN as a significant threat to coral fitness and survival, and consequently the resilience of coral reef ecosystems.

ALAN has also been shown to impact the physiology and fitness of reef fishes at various life stages, with research demonstrating significant effects of ALAN on metabolism [33,34], embryo quality [35], and post-settlement growth and survival [36,37]. As size is linked to probability of survival in the larval and post-settlement stages, with larger fish of the same age having significantly higher survival [38,39], impacts of ALAN on the size and growth in the early life stages of reef fishes may have carry-over effects for fitness and survival in later life-stages. For example, O'Connor *et al*. [36] found juvenile convict surgeonfish, *Acanthurus triostegus*, grew faster and were heavier under ALAN conditions, probably owing to increased time spent foraging under ALAN. However, the faster-growing fish under ALAN conditions had a significantly reduced probability of survival in the first 10 days post-settlement, in both the absence and presence of predators. By contrast, a long-term *in situ* study found long-term exposure to ALAN significantly reduced the growth of juvenile orange-fin anemonefish, *Amphiprion chrysopterus* [37], however, this study similarly found significantly reduced survival rates of the juvenile fish under ALAN conditions. Although the mechanisms behind the observed changes in growth under ALAN in juvenile reef fishes by both O'Connor *et al*. [36] and Schligler *et al*. [37] are unknown, the differences in findings are probably the result of many complex interacting physiological, behavioural and ecological factors. These studies both highlight the need for a greater mechanistic understanding of the impacts of ALAN on reef fishes and provide congruent evidence that the net result of ALAN exposure for juvenile coral reef fishes is decreased survival.

Many lower trophic level reef fishes and invertebrates (including the examples above), are sessile or highly site attached in juvenile and/or adult stages, and are therefore likely more susceptible to physiological impacts from ALAN as they cannot move away from artificial light in their environment. However, ALAN can also directly affect higher level consumers with greater mobility by influencing physiological responses, such as increased stress or higher metabolic rate, or indirectly through changes in resource availability and predation pressure (bottom-up and top-down effects, respectively) [20,40]. Although there has yet to be any research in the marine environment exploring how these direct impacts of ALAN on individual physiology can scale up to affect populations and/or reef communities, effects on individual growth, reproduction, and survival of organisms across trophic levels suggests that the impacts of ALAN on physiology could fundamentally alter the composition and functioning of reef communities.

### (c) Biological timings

One of the most significant ways in which ALAN can impact species on land and under water is by disrupting the timing of key biological events [3]. Many critical biological events in the ocean are highly attuned to daily, lunar, and seasonal changes in light produced by the reliable movements of the Earth and Moon around the Sun. Therefore, the addition of ALAN to an environment can mask or imitate natural light cues, resulting in an ALAN driven mismatch in life cycle timing.

#### (i) Spawning synchrony

Many marine organisms use natural light cues to align their spawning with lunar and/or seasonal cycles. Perhaps the largest and most well-known biological event orchestrated by the lunar cycle is the synchronized mass spawning of hundreds of coral species [41,42]. More than 80% of Scleractinian corals are broadcasting spawners (i.e. organisms that release eggs and sperm into the water column; [43]), and the majority of coral species use synchronized mass spawning around the full moon to maximize their fertilization success and ensure optimal environmental conditions for the dispersal, development and recruitment of coral larvae [43,44]. Recent research suggests it is the timing of moonrise, or rather the period of darkness between sunset and moonrise that occurs after the full moon, which is the cue that triggers this synchronized spawning event [45].

Furthermore, most reef fishes (e.g. [46–48]), and many other marine invertebrates from temperate and tropical regions, including sea urchins (e.g. [49]), gastropods (e.g. [50]) and bivalves (e.g. [51]), are also known to spawn with lunar or semilunar periodicity (i.e. once or twice per lunar month; [12]). Synchronizing spawning with the lunar clock may have evolved as a way for broadcast spawners to increase their probability of fertilization success [47,52]. Alternatively, and for non-broadcast spawners, aligning spawning with particular times of the lunar month, and thus intensity of nocturnal illumination, may be a strategy that minimizes risks and/or maximizes rewards to parents and/or their offspring [46,53]. By timing reproductive effort with different stages of the lunar cycle, spawners may be ensuring optimal conditions for offspring growth and settlement, and thus facilitating offspring survival at later life-stages [11].

By masking the natural light cues from the lunar cycle that marine organisms rely on to time spawning events, ALAN can suppress spawning (e.g. in the coral *Dipastrea specisosa;* [45]), or delay the gametogenesis cycle (e.g. *Acropora millepora* and *Acropora digitofera*; [32]), leading to desynchronized gamete release of broadcast spawners such as corals. Desynchronization of spawning events reduces the probability of fertilization [54], and could reduce the supply of new recruits to the population. For brood spawners that do not rely on mass synchronized spawning events for successful reproduction, a delay or disruption to the timing of spawning could lead to disrupted timing of hatching, dispersal and settlement. This disrupted timing, and dealignment of key events with the optimal lunar phase, could influence the probability of survival at these vulnerable life stages, and have significant long-term fitness consequences for individuals that do survive.

#### (ii) Early life stages

ALAN can further impact the reproductive fitness of brood spawners through masking the natural light cues used by their offspring. For example, embryos of many demersal reef fishes, particularly Pomacentrids, have evolved to hatch just after sunset [52]. This consistent timing of hatching is probably a strategy to reduce the risk of predation, as fewer planktivorous predators are active at night in coral reef environments [52,55]. However, ALAN can eliminate natural darkness at night, thus masking the cue needed to trigger hatching in reef fishes. In a laboratory experiment, embryos of the common clownfish, *Amphiprion ocellaris*, failed to hatch when exposed to low levels of ALAN (approx. 15 lux; [56]), and light intensities as low as 0.03 lux have been shown to inhibit hatching of sergeant major, *Abudefduf saxatilis*, embryos [55]. For fish larvae that do hatch and survive the pelagic larval period, recruitment back to coral reef habitat is also often synced to the lunar cycle. In fact, on coral reefs, most organisms settle disproportionately on the new moon—under cover of darkness [53,57,58]. Darkness during the new moon hinders the hunting ability of many reef-based predators [52,59], and triggers the emergence of demersal zooplankton [60], a potential prey resource for settling larva. Therefore, the intensity of light present at night can facilitate or hinder offspring survival at this vulnerable stage, through modifying predation pressure and feeding opportunities upon return to the reef, however, currently no studies have assessed how ALAN interferes with settlement and subsequent recruitment of reef fishes *in situ*.

#### (iii) Diel activity patterns

Daily and lunar light rhythms also play a critical role in shaping activity patterns and processes such as predation, in coral and temperate reef communities. Activity patterns of diurnal and nocturnal species decrease or increase, respectively, with the onset of darkness to maximize benefits and/or minimize risks associated with the changing light environment. Thus, the diel light/dark cycle mediates competition and predator–prey interactions between diurnal and nocturnal species. Similarly, predation and foraging rates of many reef organisms increase or decrease with intensity of nocturnal illumination. For example, herbivores, such as sea urchins (*Echinometra viridis* and *Echinometra lucunter*, [61]) and oysters (*Crassostrea gigas,* [62]) have been shown to increase foraging and filtration activity at night during the new moon, under the cover of darkness, whereas evidence of increased catch rates and activity patterns suggests piscivorous fishes increase feeding activity with both the full and new moon [63–65]. The ability to perceive minute changes in dim nocturnal light suggests that these organisms are susceptible to impacts from ALAN. In fact, ALAN has been shown to disrupt the daily rhythms of the Pacific oyster, *C. gigas*, by increasing valve activity with ALAN intensities as low as 0.1 lux [66]. Similarly, the gaping activity (i.e. the opening and closing of valves) of the blue mussel, *Mytilus edulis*, has been shown to decrease under white and red ALAN [67]. As light is the natural environmental cue used to regulate individual behaviours that yield these diel patterns in activity, the addition of ALAN to the reef environment can decouple activity patterns from their temporal niche, creating novel predator–prey and competitive interactions between diurnal and nocturnal species. We explore the consequences of this temporal niche shift further in §3 (see §3a, The night light niche and landscape of fear).

### (d) Movement

Many mobile marine organisms, including marine larvae, are attracted to light [68]. Small fishes, invertebrates and/or plankton will move towards a light source, through positive phototaxis, disorientation or curiosity [69], and larger fishes and invertebrates from higher trophic levels are either attracted to the light itself, or the abundance of prey it attracts [70]. Many marine organisms also exhibit negative phototaxis, and will move away from a light source, if possible (e.g. [36,71]). For example, as both a releasing and directional stimulus ALAN can increase the abundance of individual amphipod species and alter amphipod community composition during the diurnal vertical migration of emergent zooplankton [72]. Amphipods are the dominant taxa within emergent zooplankton, which is an important food source for nocturnal planktivorous predators (e.g. corals, crinoids, fishes; [73]). The presence of ALAN is also known to attract both small shoaling fishes (less than 100 mm total length) and large-bodied predators (greater than 500 mm total length; [70]). This attraction and repulsion to light means ALAN can directly alter the composition of a nocturnal reef community, where the abundance of positively phototactic species will increase and negatively phototactic species will decrease, creating both unnatural bottom-up and top-down regulation of reef ecosystems.

Phototactic behaviour can be seen in marine organisms across life-stages. Many marine fishes and invertebrates have photo receptors and exhibit behavioural responses to light in the larval settlement stage. Therefore, ALAN has the potential to influence movement and settlement patterns of organisms that are sessile or site attached as adults, including habitat-forming species such as corals (e.g. [74,75]), oysters (e.g. [76]), barnacles (e.g. [77,78]), and polychaetes [79], as well as settlement stage larval reef fishes (e.g. [36,80]). For example, a manipulative experiment showed that ALAN significantly altered the composition of temperate epifaunal assemblages, with decreased abundances of the colonial ascidian *Botrylloides leachii* and the hydroid *Plumularia setacea* under ALAN treatments. By contrast, polychaete worms *Spirobranchus lamarcki*, the copepod *Metis ignea* and amphipods from the genus *Corophium* spp were more abundant on ALAN exposed habitats [81]. Furthermore, larvae of several coral species are known to respond to light intensity (*Goniastrea aspera*, *Acropora tenuis, Ac. millepora, Oxypora lacera, Pseudodiploria strigose*) and/or wavelength (*Goniastrea favulus*, *Montipora peltiformis, Ps. strigose, Ac. millepora*) during settlement [74,75], and the presence of ALAN has been shown to reduce settlement success of key habitat-forming species (e.g. corals, *Stylophora pistillata* [82]; Pacific oyster, *Magallana gigas* [76]; barnacles, *Semibalanus balanoides* [78]*, Notochthamalus scabrosus* and *Jehlius cirratus* [77]). Interestingly, many coral reef fishes that are known to settle during the new moon (i.e. under darkness) have also shown positive phototactic behaviour in their larval settlement stage in response to intense light (e.g. light traps are used extensively for sampling reef fish larvae; [80]). This emphasizes the need for a greater understanding of how light intensity influences movement of settlement stage reef organisms, as the presence of ALAN could disrupt the natural light cues used to make informed decisions at settlement, fundamentally altering the habitat structure and community composition of both tropical and temperate reefs.

## 3. Novel ecosystems and ecosystem dynamics under artificial light at night

Reef ecosystems, like most complex food webs, are shaped by a dynamic interaction between bottom-up and top-down forces [83]. Consequently, impacts of ALAN on individual species and/or community dynamics can lead to significant ecosystem scale changes. Here, we present potential scenarios where the presence of ALAN could fundamentally alter system-level dynamics, possibly even creating novel ecosystems.

### (a) The night light niche and landscape of fear

Temporal niche partitioning between competitors and between predators and their prey is a mechanism of coexistence in some ecological communities [84]. The addition of anthropogenic light to the nocturnal environment creates what has been termed a ‘night light niche'—when diurnal species remain active at night around artificial light (initially described by Garber [85]). This change in activity could result from direct impacts of ALAN on organism physiology (e.g. suppression of melatonin production), from ALAN masking the natural light cues that organisms normally respond to (i.e. darkness, or mimicking full moon), and/or a behavioural response to take advantage of new foraging opportunities. Regardless of the mechanisms, this exploitation of a new temporal niche can result in new predator–prey and competitive interactions between diurnal and nocturnal species that do not typically interact. Furthermore, this temporal niche shift can alter the strength of existing interactions, through the effects of ALAN on different species physiology and behaviour (e.g. increase efficiency of visual predators or decrease escape response of prey owing to stress), and/or the duration of interactions (and thus the outcomes), as ALAN extends the time that diurnal species remain active.

The night light niche could also provoke non-consumptive effects—changes in behaviour or physiology in prey species as a result of the perceived increased threat of predation under ALAN—i.e. changing the landscape (or seascape) for fear of nocturnal foragers. The presence of ALAN might be expected to influence the perceived predation risk for nocturnal foragers by affecting both their ability to detect approaching predators and the visual abilities of the predators themselves. This augmented landscape of fear can alter the behaviour of reef organisms in ways that we might not predict by considering the direct impacts of ALAN alone. For example, Manríquez *et al*. [86] found feeding rates of the nocturnal Chilean abalone, *Concholepas concholepas*, were three to four times lower when exposed to ALAN, and abalone were significantly more likely to be hiding in a dark refuge compared to control conditions. Therefore, while ALAN may not lead to increased predation of this species, enhanced vigilance (i.e. refuge-seeking) under ALAN reduces time allocated to foraging, and this may have long-term impacts on fitness and productivity.

The night light niche created by ALAN in shallow marine environments could therefore create novel ecosystem dynamics by (i) augmenting the amount of time visual predators/herbivores can spend foraging, increasing the duration of their activity and thus top-down pressure on the reef; (ii) creating novel interactions between diurnal and nocturnal organisms, thus altering competition and predator–prey relationships; and (iii) altering the landscape of fear for nocturnal foragers. These novel ecosystem dynamics have simultaneous costs and benefits for reef organisms, as ALAN can both increase growth and fitness of individuals if sufficient prey resources are available to support increased foraging activity, and negatively affect physiology, reproduction and survival of organisms through altered consumptive and non-consumptive effects. Therefore, ALAN and the night light niche can shift the balance of trade-offs for reef organisms, creating novel community and ecosystem dynamics.

### (b) Species and functional diversity collapse

ALAN could cause a collapse in species or functional diversity, by imposing selective pressures on species traits that limit fitness consequences for reproduction, recruitment and survival under ALAN conditions. For example, on coral reefs, through desynchronization of spawning, ALAN can alter the composition and function of coral communities. In a recent study that examined the timing of spawning in the five most abundant coral species in the Red Sea (*Acropora eurystoma, Galaxea fascicularis, Platygyra lamellina, Dipsastraea favus and Dipsastraea favus*), Schlesinger *et al*. [44] found three of the five coral species showed no consistent, synchronized spawning pattern relative to moon-phase, temperature or wind-speed—spawning had become out of sync. When coral recruits were assessed, despite high annual recruitment rates overall, no new recruits or juveniles from two of the three impacted coral species were detected in surveys, whereas the two coral genera with the highest number of recruits (*Stylophora* and *Leptastrea*) include species that are brooders and do not employ broadcast spawning (i.e. employ internal fertilization). Ultimately, a breakdown in spawning synchrony will reduce the probability of successful coral fertilization, resulting in a potentially insufficient supply of new recruits to sustain a stable population in the long term, and coral communities may shift towards dominance of species with life-history traits that make them less vulnerable to destabilization of environmental cues, such as brooding coral species.

While the authors hypothesized the desynchronization in this study is a result of changing temperature regimes, light pollution has the potential to comparably interfere with spawning patterns by masking the lunar cues used to synchronize mass spawning events. Furthermore, as different coral species possess different tolerances to changes in the light environment [27], ALAN can further shift the balance of ‘winners' and ‘losers' in coral communities through its direct influence on settlement patterns and impact on physiological stress and symbiotic relationships of some species, and indirectly through changes to competitive pressure for space (increased growth of turfing and macroalgae) and altered grazing pressure from primary consumers. This shift in coral community composition is likely to result in a loss of coral diversity and an overall reduction in coral cover. Loss of coral diversity and cover will have implications for the physical structure of the reef, which influences the composition, diversity and functioning of the reef community it can support. Changes in the physical structure of the reef can also directly impact ecosystem services—benefits provided to humans—through altering the capacity of reefs, for example, to protect coastal assets and support fisheries.

### (c) Altered source-sink dynamics of marine metapopulations

Since many marine organisms are broadcast spawners and/or undergo a pelagic larval dispersal phase where larvae disperse in the open ocean for several days, weeks or months before settling back to a reef [87], reduced reproductive fitness from ALAN exposure at one site may not directly affect the recruitment and population demographics at that same site. As a result, the presence of ALAN at one site has the potential to alter the supply of larvae to neighbouring reefs, sometimes 10–100s of km away, even if those downstream reefs are not exposed to ALAN themselves. Similarly, the presence of ALAN on a reef can influence whether incoming larvae from distant reefs will settle (e.g. [76–78,82]), as well as their probability of post-settlement survival and recruiting to the population (e.g. [36,37]). Therefore, through direct impacts to spawning and reproduction, settlement, and post-settlement survival, ALAN could create or alter the ‘source' and ‘sink' dynamics of connected populations. Altered source-sink dynamics could alter connectivity within a metapopulation, with potentially significant implications for the stability and resilience of local populations. Furthermore, if the impacted organisms are habitat-forming species or ecosystem engineers (e.g. corals, kelp, shellfish, barnacles), ALAN can fundamentally alter the structure of the reef and habitat availability, further impacting source–sink dynamics of the supported reef species. One way ALAN may lead to a population sink is through creation of an ecological trap—a habitat that animals prefer or equally prefer, but where their fitness is lower relative to other available habitats [88]. As many species of coral reef fishes are attracted to light, particularly in their larval settlement stage [80], reefs exposed to light pollution could experience an increased influx of settling larvae, which could contribute to reef resilience. However, as light at night can also reduce individual fitness of settlement stage larvae through physiological changes [36,37], enhanced predation risk [36] and increased top-down pressure from attraction of large predators [70], the attraction of larval reef fish to light-polluted habitats could be creating an ecological trap, significantly altering recruitment dynamics on tropical reefs and population persistence of vulnerable species.

### (d) Disruption of diel vertical migrations

The largest animal migration on the Earth is the diel vertical migration (DVM) of mesopelagic zooplankton from deeper waters during the day to near-surface waters during the night to feed [89]. Migrating zooplankton are followed by their predators, producing complex predator–prey migration patterns across multiple trophic levels [89]. This large-scale migration, which occurs in all oceans at all latitudes (e.g. [90]), plays a key role in the flow of energy and material between oceanic zones, and in biogeochemical cycles including carbon sequestration [91,92], and hence buffering global climate change [89]. DVM behaviour is generally controlled by ambient irradiance, with illumination from above inhibiting movement, and the setting sun, and thus a reduction in surface light levels, triggering an upward migration. The migrating zooplankton reach their shallowest distribution on the darkest nights of the lunar cycle, i.e. during the new moon, whereas the full moon is known to suppress the upward movement of all trophic levels [11]. Because of the nuanced relationships between ambient light levels in the ocean and the vertical movement of zooplankton and higher trophic level predators, ALAN can interfere with and disrupt the timing of the diel migration patterns of key organisms. Ludvigsen *et al*. [93] have shown that light from large vessels, or even from headlamps on a small open vessel, introduce enough light pollution to induce an avoidance response in the pelagic community down to more than 80 m depth. In addition to interfering with the initiation of zooplankton migration, ALAN can mediate changes in DVM through changing the behaviour of higher trophic organisms (i.e. increased/decreased consumption rates), through changes in species distributions (e.g. [72]), and through shifts in trophic dynamics. Impacts of ALAN on DVM will also probably affect carbon transport and biogeochemical cycles, with consequences to the functioning and connectivity of marine systems. As a result, disruption to DVM patterns from ALAN could have widespread consequences for the marine environment, potentially creating entirely novel ecosystems. However, attempts to quantify such impacts will need to consider sampling methods, as any nocturnal sampling method that introduces artificial light into the sampling environment will alter outcomes [93].

## 4. Future directions and conclusion

This paper demonstrates that ALAN can impact all levels of biological organization on temperate and tropical reefs, from individuals to community and ecosystem functioning, however, it also highlights clear gaps in evidence that hinders our understanding of the extent of the impacts of ALAN in marine systems. We present several key knowledge gaps below, where we suggest future research on ALAN on reef systems should be directed.

### (a) Multiple stressors

Increasingly, the marine environment is under threat from numerous stressors associated with human activities (e.g. chemical and noise pollution, ocean warming, acidification and de-oxygenation) [94]. These stressors more often than not co-occur with ALAN in space and time, making it challenging to assess the isolated impacts of ALAN in natural systems. Despite this, very few studies have actually considered the effects of ALAN alongside other co-occurring stressors in the marine environment [94–96]. Without empirical data, it is difficult to predict the combined effects of ALAN and multiple other stressors, as individual stressors may have species specific impacts, can impact different sensory systems (e.g. visual (light pollution), auditory (noise pollution) and olfactory (chemical pollution)) within individuals (e.g. [97]), and can have interactive, additive or antagonistic effects [98]. For example, observed impacts of ALAN on population dynamics and their consequences to ecosystem functioning are likely to be exacerbated with ocean warming, since thermal stress and ALAN similarly affect photosynthesis in primary producers [99], alter microbial communities [100] and alter growth and fitness of reef fishes (e.g. [101]), resulting in functional changes of systems [102]. This is probably true for corals, where increasing temperatures and ALAN similarly increase coral stress and mortality, and negatively influence the timing of gametogenic and spawning cycles [42–44], with potential for severe impacts to coral reproduction success and, consequently, overall diversity on a reef. However, the impacts of ALAN and ocean warming on sea urchins are probably antagonistic. Through grazing, sea urchins play a key role in the top-down control of macroalgal abundance on temperate reefs [103,104], and in high abundances, urchins can create barrens—large areas devoid of habitat-forming macroalgae [105,106]. While increased temperatures has been shown to increase urchin consumption rates [107], ALAN is predicted to decrease consumption rates of nocturnal urchins (e.g. through a seascape of fear). Likewise, these stressors can affect the kelp themselves, through e.g. changes in the kelp-associate microbiota [108] and their photosynthetic rates [109]. Such changes can not only have consequences to local primary productivity, but also influence the chemical defences of algae and their nutritional value, impacting interactions with herbivores, such as sea-urchins. At the community and/or ecosystem level, these interacting, additive or antagonistic stressors will probably create clear ‘winners' and ‘losers', which will ultimately determine the net effects of these stressors. More studies assessing combined effects of ALAN and other co-occurring stressors are therefore imperative to advance our knowledge and guide future management actions.

### (b) Impacts of long-term exposure to artificial light at night

Our current understanding of how tropical and temperate reef organisms respond to ALAN is derived from short-term experimental or observational studies, spanning days to months in duration, and all within a single generation. The lack of long-term, multi-generational studies presents a significant gap in our understanding of how organisms may alter their behaviour, activity and/or physiology to maintain function under chronic ALAN exposure, and how ALAN might shift the selection pressure on certain phenotypic or genotypic traits. Though challenging, longer-term and multi-generational studies are necessary to determine whether phenotypic plasticity, parental effects or evolutionary compensation mechanisms allow marine populations to adapt to novel light environments. These questions can be addressed through laboratory-based multi-generational studies, as has been done for other environmental pollutants and climate stressors (e.g. [110–112]).

### (c) Latitudinal differences in artificial light at night sensitivity

Interestingly, most of the research on the impacts of ALAN on subtidal reefs has been on tropical (e.g. corals and coral reef fishes) and subtropical (e.g. sea urchins) reef organisms, whereas many of the studies we have included in this synthesis on ALAN in temperate regions have focused on intertidal organisms (e.g. barnacles, shellfish and microphytobenthos). Intertidal communities experience very different environmental conditions than subtidal reef communities, including likely exposure to higher ALAN intensities; however, we have had to rely on these studies as research on the impacts of ALAN in subtidal temperate reefs is still minimal. This difference in research focus between tropical and temperate marine habitats is potentially owing to the fact that coral reefs are more likely to be vulnerable to impacts of light pollution. Water clarity on tropical reefs is typically much greater than in temperate marine habitats, and thus light will penetrate deeper through the water column and is more likely to reach the reef. This difference in light environments at different latitudes could potentially also result in a stronger reliance on nocturnal illumination as a cue for many critical biological events and processes on tropical reefs, whereas variation in moonlight in higher, temperate, latitudes may not be as important a cue as the more considerable changes in daylength or water temperature, both of which are more variable in temperate environments. Alternatively, the predictable but more annually variable lunar irradiance in temperate regions (e.g. [113]) means organisms can determine the specific time of year and month using lunar cues, and therefore temperate species may be equally or more sensitive to small changes in light environment as their tropical counterparts. This synthesis highlights the need for more research on temperate reefs to determine if and how the sensitivity of temperate reef organisms and trophic interactions to ALAN differ from those at lower latitudes.

## 5. Conclusions

Research on the impacts of ALAN on tropical and temperate reef organisms is growing, and mounting evidence suggests ALAN has the potential to significantly alter community dynamics and trophic interactions, and disrupt ecosystem functions. Consequently, these impacts will have flow on effects for ecosystem services and human economies. However, our understanding of the overall impacts of ALAN to reef systems remains insufficient, and large knowledge gaps need to be addressed to facilitate management and mitigation measures for ALAN on tropical and temperate reefs. This research is sorely needed, as light pollution is largely an unregulated environmental stressor globally, with clear ecological impacts both on land and in the ocean.

## Data Availability

This article has no additional data.
